# The influence of distal-end heat treatment on deflection of nickel-titanium archwire

**DOI:** 10.1590/2177-6709.21.1.083-088.oar

**Published:** 2016

**Authors:** Marcelo Faria da Silva, Célia Regina Maia Pinzan-Vercelino, Júlio de Araújo Gurgel

**Affiliations:** 1 Private practice, Palmas, Tocantins, Brazil; 2 Assistant Professor, Universidade Ceuma, Department of Orthodontics, São Luís, Maranhão, Brazil

**Keywords:** Orthodontic wires, Heat treatment, Mechanical properties

## Abstract

**Objective:**

The aim of this *in vitro* study was to evaluate the deflection-force behavior of nickel-titanium (NiTi) orthodontic wires adjacent to the portion submitted to heat treatment.

**Material and Methods:**

A total of 106 segments of NiTi wires (0.019 x 0.025-in) and heat-activated NiTi wires (0.016 x 0.022-in) from four commercial brands were tested. The segments were obtained from 80 archwires. For the experimental group, the distal portion of each segmented archwire was subjected to heat treatment (n = 40), while the other distal portion of the same archwire was used as a heating-free control group (n = 40). Deflection tests were performed in a temperature-controlled universal testing machine. Unpaired Student's t-tests were applied to determine if there were differences between the experimental and control groups for each commercial brand and size of wire. Statistical significance was set at *p* < 0.05.

**Results:**

There were no statistically significant differences between the tested groups with the same size and brand of wire.

**Conclusions:**

Heat treatment applied to the distal ends of rectangular NiTi archwires does not permanently change the elastic properties of the adjacent portions.

## Abstract (pt)

**Objetivo:**

o objetivo dessa pesquisa *in vitro* foi avaliar a deflexão na porção adjacente à área do tratamento térmico realizado no extremo distal de fios ortodônticos de níquel-titânio (NiTi).

**Métodos:**

foram testados 160 segmentos de fios de NiTi (0,019" x 0,025") e NiTi termoativado (0,016" x 0,022") de quatro marcas comerciais; obtidos de 80 arcos. No grupo experimental (n = 40), realizou-se o tratamento térmico no extremo distal; enquanto no grupo controle (n = 40), o extremo distal foi mantido isento de tratamento térmico. Os testes de deflexão foram realizados em máquina universal de ensaios, com controle de temperatura. Foi aplicado o teste *t* de Student não-pareado, visando observar a diferença entre os grupos experimental e controle, em cada espessura e marca. A significância estatística foi fixada com valor de*p* < 0,05.

**Resultados:**

não foram encontradas diferenças estatisticamente significativas entre os grupos testados, de mesma espessura e marca comercial.

**Conclusão:**

conclui-se que o tratamento térmico não influenciou na deflexão da região adjacente à extremidade de fios de NiTi submetidos ao tratamento térmico.

## INTRODUCTION

Nickel-titanium (NiTi) wires are commonly used in the early stages of orthodontic treatment because they release light constant forces that are appropriate for alignment and leveling. Among the types of NiTi wires, heat-activated NiTi wires stand out because their action is influenced by temperature.^1^ These types of wire exhibit crystallographic transformation under controlled temperatures. However, the austenite finish temperature (Af) of heat-activated NiTi wires has shown variability among commercial brands.^2^

NiTi archwires are used for the initial leveling phase of orthodontic treatment performed with conventional or self-ligating brackets. Self-ligating systems reduce friction, thereby allowing shifting, especially of thin or rectangular wires.^3^ This may result in incisor protrusion and/or in protruding wire in the distal portion of molar tubes. This last fact may cause impingement of the oral tissue surrounding the distal portion of molar tubes. To minimize this injury, crimpable stops are recommended, as they are placed over the archwire in any interbracket spaces so as to limit the shift of archwires.^3 ^Another clinical procedure to limit archwire shifting is to heat the ends of archwire, allowing it to become formable. At room temperature, heat treatment causes reduction in stiffness of metal alloys. Excessive heating of the wire leads to loss of tempering of the alloy and facilitates bending as a consequence of changes caused to the mechanical properties of the wire. Clinical orthodontists usually carry out heat treatment with a blowtorch or a conventional cigarette lighter. For NiTi wires, heat treatment at ± 650 °C causes loss of mechanical properties.^4^

Following heat treatment in the distal ends, the wires are cinched back to the lingual or gingival sides. However, this effective and easy-to-perform technique requires scientific validation due to influences caused to the mechanical properties of the adjacent segment of the treated archwire.

Studies have shown that exposure of NiTi wires to temperatures of 500 °C and 600 °C alters their mechanical properties, reducing the force released in deflection tests.^4^^,^ 5 ^,^ 6 Because metal alloys are good thermal conductors, it is understood that tempering orthodontic wires leads to heat dissipation at other portions of the archwire. Thus, heat treatment of the distal end of NiTi wires is a matter of concern because it may negatively affect deflection of the area adjacent to heat treatment, which might include molar and premolar regions.

This *in vitro* study analyzed the force present in different commercial brands of NiTi wires in a deflection test. The null hypothesis was that heat treatment of the distal ends of NiTi orthodontic archwires does not alter deflection in the adjacent area.

## MATERIAL AND METHODS

Sample size was calculated using Gpower^TM^, version 3.1 software (University of Düsseldorf, Düsseldorf, Germany). The parameters adopted were the following: significance level α of 5%; test power of 70%; and effect size equal to 1. A sample size (n) of 10 specimens in each group was determined. The following archwires were used in this study: 40 NiTi heat-activated archwires from the same lot with cross sections of 0.016 x 0.022-in and 40 NiTi arches from the same lot with cross sections of 0.019 x 0.025-in (Table 1). Thus, four brands were selected for evaluation, with one rectangular cross section evaluated for each type of NiTi archwire.

**Table 1:** - Complete description of specifications of the nickel-titanium (NiTi) wires tested.

**Groups**	**Commercial name**	**Manufacturer**	
0.016 x 0.022-in	E	Thermal*	Shenzhen Superline Technology, Shenzhen, China.
			(distributed by Eurodonto^TM^, Paraná, Brazil)
	M	Thermo Plus*	Dental Morelli^TM^, Sorocaba, São Paulo, Brazil
	O	Flexy Thermal Smart 37^o^C	Beijing Smart Technology^TM^, Beijing, China.
			(distributed by Orthometric^TM^, Marília, São Paulo, Brazil)
	U	Nitinol heat-activated*	3M^TM^/Unitek^TM^, Monrovia, California, USA.
0.019 x 0.025-in	E	Super Elastic	Shenzhen Superline Technology^TM^, Shenzhen, China.
			(distributed by Eurodonto^TM^, Paraná, Brazil)
	M	Super Elastic	Dental Morelli^TM^, Sorocaba, São Paulo, Brazil
	O	Flexy Super Elastic	Beijing Smart Technology^TM^, Beijing, China.
			(distributed by Orthometric^TM^, Marília, São Paulo, Brazil)
	U	Nitinol Superelastic	3M^TM^/Unitek^TM^, Monrovia, California, USA

*No information about the transformation temperature is disclosed on the package.

The samples were obtained by cutting 28 mm of both straight distal portions of each archwire. The segment without heat treatment comprised the control group. In the experimental group, heat treatment was carried out and resulted in distal segment annealing. Initially, a 5-mm length was selected at the end of each segment, and heat treatment was performed for three seconds, with the aid of a miniature gas orthodontic blowtorch (Ortho-Gas GB-2001, Blazer S.A., Guangzhou, China), causing the wire to become red hot. The duration of heat treatment, the flame and the temperature were all calibrated. The flame was standardized by a blue color measuring 20 mm in height. Flame temperature was controlled with the aid of a portable temperature gauge (Termopar, Novus - Smart Meter, Porto Alegre, Rio Grande do Sul, Brazil), so as to stabilize temperature at 650 °C. All specimens comprising the experimental group were positioned over the flame, as described above, until it acquired a red-hot color, indicating that annealing had been achieved. The samples were placed in plastic containers and labeled for test operator blinding. Thus, 80 specimens from the control group and 80 specimens from the experimental group were generated.

A three-point bending test was carried out for the specimen wires (n = 10). All samples were loaded under the same protocol and according to ISO/CD 15841.^7^ A metallic device consisting of two 5-mm diameter pins welded vertically 14 mm apart was used. A maxillary premolar metallic bracket was attached to one of the pins, and a simple tube specifically designed for maxillary molars was attached to the other pin. Both attachments had a 0.22 slot without torque and angulation (Morelli^TM^, Sorocaba, São Paulo, Brazil).

Each specimen was placed under the bracket and the attachment so that the distance between the distal end of each bracket and the wire was the same on both sides. The experimental wires were attached to the bracket by gray elastic ligature in modules (Morelli^TM^, Sorocaba, São Paulo, Brazil). Then, the test device was adapted to a universal testing machine inside a thermal chamber (EMIC^TM^, São José dos Pinhais, Paraná, Brazil) at a temperature of 36 °C.

Each wire was first loaded to a deflection of 3.1 mm (activation) and then unloaded (deactivation) at a rate of 1 mm/min with a load cell of 50 N (EMIC^TM^, Model DL 2000, São José dos Pinhais, Brazil). Both force and deflection (activation and deactivation at 1.0 mm) were compared. 

Statistical analysis was performed by Statistica version 5.1 software (StatSoft Inc., Tulsa, USA) to examine the effect of heat treatment on the specimens of the same wire type. Data were examined for normality with the Kolmogorov-Smirnov test. The mean values for activation and deactivation were analyzed by t-test to test for a significant difference after heat treatment (*p* < 0.05).

## RESULTS

The values achieved by the 0.019 x 0.025-in wires during activation and deactivation were higher than those for the smaller-diameter (0.016 x 0.022-in) wires. This effect was due to differences in cross-section and stiffness.

Deflection force at activation and deactivation of 1 mm did not show significant difference among wires, with similar cross-sections for either the experimental or control groups (*p* > 0.05) (Table 2). 

**Table 2:** - Force (Newton) for activation and deactivation measured at 1 mm deflection for control and experimental samples (0.016 x 0.022-in heat-activated NiTi).

**Wire**	**Cross section**	**Activation**	**Deactivation**				
		**Control**	**Experimental**	***p* value**	**Control**	**Experimental**	***p* value**
E	0.016 x 0.022-in	7.50 (0.65)	6.60 (1.99)	0.186	1.81 (0.31)	1.63 (0.68)	0.456
M	0.016 x 0.022-in	7.16 (0.44)	7.09 (0.54)	0.761	2.24 (0.19)	1.96 (0.46)	0.096
O	0.016 x 0.022-in	5.63 (0.41)	5.60 (0.44)	0.877	1.30 (0.36)	1.24 (0.36)	0.737
U	0.016 x 0.022-in	5.84 (0.37)	5.98 (0.76)	0.633	1.23 (0.24)	1.36 (0.29)	0.293

*Significance set at 5%.

**Table 3:** - Force (Newton) for activation and deactivation measured at 1 mm deflection for control and experimental samples (0.019 x 0.025-in NiTi).

**Wire**	**Cross section**	**Activation**	**Deactivation**				
		**Control**	**Experimental**	***p* value**	**Control**	**Experimental**	***p* value**
E	0.019 x 0.025-in	14.31 (0.46)	14.09 (0.81)	0.457	5.62 (0.32)	5.49 (0.31)	0.361
M	0.019 x 0.025-in	15.18 (0.92)	15.14 (0.80)	0.920	7.44 (0.44)	7.60 (0.51)	0.470
O	0.019 x 0.025-in	12.83 (1.71)	13.64 (0.65)	0.175	5.44 (0.49)	5.27 (0.18)	0.306
U	0.019 x 0.025-in	14.32 (0.89)	13.29 (2.72)	0.269	6.24 (0.65)	6.43 (0.34)	0.422

*Significance set at 5%.

The four commercial wire brands showed mean deactivation forces of 1.6 N for the 0.016 x 0.022- in wires and 6.2 N for the 0.019 x 0.025-in wires.

In general, for the 0.016 x 0.022-in wires, it was observed that both activation and deactivation values were lower in the experimental group than in the control group (Table 2). An exception to this rule was observed for the "U" wires which showed higher mean values in the experimental group (5.98 N) compared to the control group (5.84 N). Additionally, during deactivation, the means for the experimental group (1.36 N) tended to be higher than in the control group (1.23 N). No significant difference was observed in these values between the control and experimental groups (*p* > 0.05) (Table 2).

Regarding 0.019 x 0.025-in wires (Table 2), during activation, only the "O" brand wires had higher mean values in the experimental group (13.64 N) compared to the control group (12.83 N). However, a similar result was not found for deactivation. For the experimental group, the "U" and "M" wires achieved values of 7.60 N and 6.43 N, respectively, and the control group achieved values of 7.44 N and 6.24 N, respectively. No significant difference was observed between the control and experimental groups (*p* > 0.05) (Table 2).

## DISCUSSION

The results of this study showed that heat treatment of the tested NiTi wires ends did not alter the deflection of the adjacent portions of orthodontic archwires. Therefore, there was no change in the deflection of NiTi wires in the region adjacent to where heat treatment had been used to cinch back the wire at the molar tube. These data reveal that heat treatment applied to the distal end of rectangular NiTi wires does not compromise the deflection force in the second premolar region.

According to the methods used in the present study, heat treatment should be performed at the final 5 mm of NiTi wires, whether conventional or heat-activated. In clinical practice, performing heat treatment in the distal end of a wire is usually a simple procedure for every orthodontist. To eliminate measurement bias in our study, we used a miniature orthodontic gas blowtorch that had been previously calibrated under controlled temperatures.

The NiTi orthodontic wires exposed to temperatures of 500 °C showed changes in some of their mechanical properties. However, when wires are exposed to temperatures of 600 °C, the properties are completely lost.^4^In this study, heat treatment was performed at a temperature of 650 °C until the wire became red-hot. The same procedure is used by orthodontists who wish to easily bend an orthodontic wire of a different alloy.

The objective of the present study was not to compare commercial brands, but to compare the effects of heat treatment on different cross-section and brands of wires. However, for Unitek^TM^/3M^TM^ 0.016 x 0.022-in wires, an insignificant increase in stiffness was observed in the experimental group. This change did not appear to be sufficient to hinder tooth movement. For the 0.016 x 0.022-in wires (Eurodonto^TM^, Morelli^TM^, Orthometric^TM)^, activation and deactivation values were lower in the experimental group compared to the control group, indicating that heat treatment caused no significant changes in adjacent regions (Table 2). Because these differences between groups were not statistically significant, it should be understood that the reduction in force necessary for deflection was not significant to orthodontic movement. 

Only during activation Orthometric 0.019 x 0.025-in NiTi wires exhibited higher mean values for the experimental group compared to the control group. Similar results were not observed for deactivation. Nevertheless, for Unitek^TM^/3M^TM^ and Morelli^TM^ wires, the experimental group showed higher values than those of the control group for deactivation. This difference would indicate that heat treatment causes a slight increase in stiffness for some NiTi wires (Table 2).

In this study, heat treatment in the distal portion of NiTi wires did not influence deflection for various tested brands. Thus, it seems clear that orthodontists can select the NiTi wire of their choice and anneal any desired spot (cinched back) to the archwire. Clinicians should prevent soft-tissue irritation caused by swiveling of the archwire; this should be the standard of care during severe anterior crowding correction. Crowding correction with NiTi wires is efficiently performed between appointments when wire excess is common in the distal region of molar tubes. For rectangular archwires, correct leveling and alignment allowed shifting of the archwire, leading not only to soft tissue irritation, but also to arch distortion and asymmetry. Although crimpable stops are the most commonly indicated methods to avoid archwire shifting, they partially retain the archwire due to interbracket distance, thus allow free space for shifting. Heating the distal ends of NiTi archwires is advantageous due to being an inexpensive, rapid and simple procedure. This fact is particularly important for treatment performed with self-ligating systems because low friction allows archwire shifting.^3^^,^ 8 ^,^ 9

Although there are many studies in the literature on the deflection of NiTi wires,^1^^,^ 4 ^,^ 10 ^,^ 11 to the best of our knowledge, no previous study has determined the influence of thermal treatment in NiTi wires or in heat-activated NiTi wires. In the future, laboratory and clinical research can examine the aspects highlighted in this study in greater detail.

Although values obtained for *in vitro* experiments do not always coincide with clinical reality, they provide a means of comparison with other studies and guide the selection of the best material for a given stage of treatment.^12^^,^ 13 The present study demonstrated that heat treatment of distal ends can be performed with heat-activated and conventional rectangular NiTi wires without any risk of detrimentally affecting alignment or leveling of teeth adjacent to the area chosen for heat treatment. 

## CONCLUSIONS

Based on the methods used in this *in vitro* study, we conclude that heat treatment does not permanently change the elastic properties of adjacent areas in conventional or heat-activated rectangular NiTi archwires.
